# Is Brazil Prepared for the Hip Fracture Epidemic in the Elderly: A National Ecological Analysis of In‐Hospital Mortality, Regional Disparities, and Hospital Costs (2008–2024)

**DOI:** 10.1155/joos/8869381

**Published:** 2026-04-01

**Authors:** Deivid Ramos dos Santos, Jean Klay Santos Machado, Eduardo Cezar Silva dos Santos, Luciano Elias Barboza, Mauricio da Camara Ferreira, Maria Clara Almeida Sadala dos Santos, Marcos Henrique de Sousa Silva, Ricarte Roberto Cruz Pantoja

**Affiliations:** ^1^ Department of Orthopedics and Traumatology, Universidade do Estado do Pará (UEPA), Belém, Pará, Brazil

**Keywords:** epidemiology, femoral fracture, mortality, older adults, oldest-old

## Abstract

**Objective:**

To analyze temporal trends, regional disparities, and the economic impact of in‐hospital mortality due to femoral fractures among older adults in Brazil between 2008 and 2024.

**Methods:**

This ecological study was based on data from the Hospital Information System of the Brazilian Unified Health System (SIH/SUS). In‐hospital mortality rates were calculated per 100,000 inhabitants and stratified by age groups (60–64, 65–69, 70–74, 75–79, and ≥ 80 years) and geographic regions. Temporal trends were assessed using simple linear regression.

**Results:**

During the study period, 45,405 deaths due to femoral fractures among older adults were recorded during the first hospital admission. The national in‐hospital mortality rate increased from 0.87 per 100,000 inhabitants in 2008 to 1.10 per 100,000 inhabitants in 2024. The ≥ 80‐year age group exhibited the highest mortality rates (mean: 60.7 ± 8.69), whereas the 60‐ to 64‐year group showed the lowest values (0.59 ± 0.36). The Southeast and South regions presented the highest age‐adjusted mortality rates, with marked regional heterogeneity and wider confidence intervals observed in the North and Northeast regions. A sustained upward trend in mortality was identified, particularly among the oldest‐old population, concomitant with a substantial increase in hospital‐related costs.

**Conclusion:**

In‐hospital mortality due to femoral fractures among older adults in Brazil demonstrates a rising trend, significant regional inequalities, and a considerable economic burden. These findings suggest that, in the context of rapid population aging, Brazil remains insufficiently prepared to address the growing epidemic of femoral fractures, underscoring the need for integrated public health policies, structured care pathways, and multidisciplinary protocols focused on geriatric trauma care.

## 1. Introduction

Femoral fractures among older adults represent one of the most severe manifestations of bone fragility associated with population aging, constituting a major challenge and a significant global public health problem, particularly in emerging countries such as Brazil [1, 2]. It is estimated that more than 1.6 million proximal femoral fractures occur worldwide each year, a number projected to reach up to 4.5 million annually by 2050, driven by accelerated population aging, especially in low‐ and middle‐income countries [2].

In Brazil, the epidemiological scenario remains incompletely understood, as there are no nationwide studies comprehensively assessing temporal trends in hospitalization rates and mortality associated with femoral fractures. As a result, national health policies are often based on extrapolations from studies conducted in other countries. The existing Brazilian studies are limited by small population samples and fail to capture the true national burden of this public health problem, particularly given the country’s vast territorial extension and pronounced economic, structural, and healthcare access disparities [3–5].

Recent studies indicate that factors such as advanced age, male sex, functional frailty, the presence of multiple comorbidities, chronic kidney disease, and postoperative infectious complications are strongly associated with mortality following proximal femoral fractures [4]. In addition, prognostic tools such as the Nottingham Hip Fracture Score (NHFS), originally developed in the United Kingdom, as well as mortality prediction models such as that proposed by Machado [6], have demonstrated good performance in predicting 30‐day mortality, thereby supporting immediate clinical decision‐making and the implementation of preoperative care strategies aimed at mitigating adverse outcomes.

In light of the lack of consolidated nationwide data on femoral fractures among older adults in Brazil, this study aims to analyze temporal trends in hospital admissions and associated mortality related to this condition. Stratification by age group and geographic region is intended to identify specific vulnerabilities within the older population, thereby providing evidence to support the development of more equitable and effective public health policies focused on prevention, timely treatment, and functional rehabilitation of this high‐risk group.

In light of the lack of consolidated nationwide data on femoral fractures among older adults in Brazil, this study aims to analyze temporal trends in hospital admissions, in‐hospital mortality, and hospital costs and economic burden associated with this condition. Stratification by age group and geographic region is intended to identify specific vulnerabilities within the older population, thereby providing evidence to support the development of more equitable and effective public health policies focused on prevention, timely treatment, and functional rehabilitation of this high‐risk group.

## 2. Methods

### 2.1. Study Design and Data Source

This retrospective ecological study was conducted using secondary data obtained from the Hospital Information System/Brazilian Unified Health System (SIH/SUS), a nationwide, open‐access administrative database that records all hospital admissions reimbursed by the public healthcare system. Data were extracted and organized from the official DATASUS platform [7]. The SIH/SUS database adopts the International Classification of Diseases, 10th Revision (ICD‐10) for diagnostic coding. In the present analysis, all hospitalizations of individuals aged 60 years and older, residing in the five Brazilian macroregions (North, Northeast, Southeast, South, and Midwest), with a primary diagnosis of femoral fracture (ICD‐10 codes S72.0, S72.1, S72.2), recorded between January 2008 and December 2024, were included.

### 2.2. Variables and Measures

The following variables were extracted from the database: (1) number of hospital admissions; (2) number of in‐hospital deaths associated with femoral fractures; (3) in‐hospital case fatality rate (%), defined as the number of deaths divided by the total number of hospital admissions for each year and geographic region; (4) total hospital expenditure, corresponding to the total amount reimbursed by SUS for these admissions; (5) mean cost per hospital admission (BRL), calculated as total expenditure divided by the number of admissions; and (6) age‐specific population‐based in‐hospital mortality rate (per 100,000 inhabitants), calculated using the corresponding annual population estimates obtained from Brazilian Institute of Geography and Statistics (IBGE) [8] as denominators, thereby standardizing mortality according to the population at risk for each year.

Population estimates were obtained from IBGE, stratified by calendar year, geographic macroregion, and age group (60–64, 65–69, 70–74, 75–79, and ≥ 80 years). The use of age‐ and region‐specific denominators enabled population‐based standardization of in‐hospital mortality rates, allowing robust comparisons across regions and age strata regardless of demographic heterogeneity. All monetary values presented in the tables correspond to nominal amounts as recorded in the official SIH/SUS database.

### 2.3. Statistical Analysis

Data were organized using Microsoft Excel (Microsoft Office 365). Descriptive statistics were calculated, including means, medians, and standard deviations. Temporal patterns were explored through graphical analyses with trend lines, and data visualization techniques were applied to illustrate trends and regional patterns over time.

The temporal evolution of the in‐hospital mortality rate was assessed using simple linear regression models, with calendar year as the independent variable. Comparisons across geographic regions and age groups were performed using visual inspection and nonparametric statistical tests, specifically the Kruskal–Wallis test, followed by Dunn’s post hoc test when appropriate. Statistical significance was set at *p* < 0.05.

### 2.4. Ethical Considerations

This study was conducted in accordance with the ethical guidelines of the Research Ethics Committee of the Universidade do Estado do Pará (UEPA) and with Resolution No. 510/2016 of the Brazilian National Health Council.

## 3. Results

### 3.1. Hospital Admission Rates

Between 2008 and 2024, a total of 918,952 hospital admissions due to femoral fractures were recorded among individuals aged 60 years and older in Brazil, corresponding to an average of approximately 54,000 admissions per year. The mean national hospital admission rate over the study period was 206.4 cases per 100,000 inhabitants. This rate increased from 176.29 to 249.79 admissions per 100,000 inhabitants, representing a 41.7% increase over the study period (Table 1).

**TABLE 1:** Hospitalizations due to femoral fracture and hospitalization admission rate (HIR) in the elderly by region and year—Brazil, 2008–2024.

Region/year	North			Northeast			Southeast			South			Central West			Total		
	H	Pop	HIR	H	Pop	HIR	H	Pop	HIR	H	Pop	HIR	H	Pop	HIR	H	Pop	HIR
2008	1177	1004936	117.12	6.304	5.159.311	122.19	18.678	8948397	208.73	6.036	3058800	197.33	1.857	1144857	162.20	34.052	19.316.301	176.29
2009	1185	1045161	113.38	6.807	5.318.918	127.98	19.431	9264982	209.73	6.367	3174141	200.59	2.057	1195837	172.01	35.847	19.999.039	179.24
2010	1099	1089024	100.92	6.230	5.481.802	113.65	19.916	9600603	207.45	6.602	3296678	200.26	2.056	1249935	164.49	35.903	20.718.042	173.29
2011	1425	1136668	125.37	7.104	5.648.113	125.78	20.800	9956436	208.91	7.003	3426502	204.38	1.965	1307398	150.30	38.297	21.475.117	178.33
2012	1539	1187647	129.58	6.893	5.820.794	118.42	21.375	10333056	206.86	7.244	3563485	203.28	2.247	1368501	164.19	39.298	22.273.483	176.43
2013	1613	1242063	129.86	7.697	6.000.720	128.27	22.112	10731315	206.05	8.113	3707969	218.80	2.304	1433496	160.73	41.839	23.115.563	181.00
2014	1916	1299803	147.41	8.477	6.187.754	137.00	23.201	11149136	208.10	8.569	3859955	222.00	2.450	1502048	163.11	44.613	23.998.696	185.90
2015	1828	1360593	134.35	8.726	6.380.848	136.75	24.126	11583471	208.28	9.416	4018702	234.30	2.878	1574004	182.85	46.974	24.917.618	188.52
2016	1880	1424371	131.99	9.264	6.579.657	140.80	27.423	12034039	227.88	10.384	4183898	248.19	3.408	1649423	206.62	52.359	25.871.388	202.38
2017	2006	1491444	134.50	10.432	6.786.474	153.72	29.091	12500599	232.72	10.429	4354776	239.48	3.696	1728597	213.82	55.654	26.861.890	207.19
2018	2331	1561723	149.26	11.096	7.004.192	158.42	30.099	12980463	231.88	11.324	4531798	249.88	4.057	1811342	223.98	58.907	27.889.518	211.22
2019	2426	1634716	148.40	12.214	7.233.983	168.84	32.098	13468867	238.31	11.655	4715523	247.16	4.569	1897672	240.77	62.962	28.950.761	217.48
2020	2263	1701969	132.96	12.186	7.459.032	163.37	32.296	13935387	231.76	11.957	4900765	243.98	4.229	1982290	213.34	62.931	29.979.443	209.91
2021	2718	1761235	154.32	13.549	7.673.443	176.57	34.834	14341669	242.89	12.804	5067640	252.66	4.592	2059130	223.01	68.497	30.903.117	221.65
2022	3326	1828263	181.92	15.457	7.905.831	195.51	38.336	14757419	259.77	14.259	5231620	272.55	5.436	2141366	253.86	76.814	31.864.499	241.06
2023	3635	1908027	190.51	16.995	8.173.561	207.93	39.320	15241645	257.98	14.954	5420769	275.86	6.141	2237489	274.46	81.045	32.981.491	245.73
2024	3927	1992741	197.07	17.396	8.467.135	205.45	40.064	15747890	254.41	15.438	5622774	274.56	6.135	2339077	262.28	82.960	34.169.617	242.79
Total	36294	24.670.384	147.12	176.827	113.281.568	156.10	473.200	206.575.374	229.07	172.554	72.135.795	239.21	60.077	28.622.462	209.89	918.952	445.285.583	206.37

*Note:* Population expressed in millions of inhabitants aged 60 years or older. H = hospital admissions; Pop = population (≥ 60 years); HIR = hospitalization (admission) rate per 100,000 elderly inhabitants.

The Southeast region accounted for the highest absolute number of hospital admissions, with 473,200 cases, representing 51.5% of all femoral fracture–related hospitalizations nationwide. This was followed by the Northeast (176,827 admissions), South (172,554), Midwest (60,077), and North (36,294) regions.

Regarding hospital admission rates standardized by the age‐specific population at risk, the South and Southeast regions exhibited the highest mean rates over the study period (239.2 and 229.1 per 100,000 inhabitants, respectively), followed by the Midwest (209.9), Northeast (156.1), and North (147.1 per 100,000 older adults).

### 3.2. In‐Hospital Deaths

During the study period, 45,405 in‐hospital deaths due to femoral fractures among older adults were recorded during the first hospital admission, resulting in an overall in‐hospital case fatality rate of 4.94%. Higher mortality was observed among the oldest‐old population. Age‐stratified analysis demonstrated a progressive increase in in‐hospital mortality, rising from 1.82% among individuals aged 60–64 years to 7.22% among patients aged 80 years and older. The oldest‐old age group accounted for 47.5% of all hospital admissions (Table 2).

**TABLE 2:** Distribution of hospital admissions, deaths, and hospital mortality rate (HMR per 100 admissions) by sex and age group in older adults with femoral fracture—Brazil, 2008–2024.

Variable	Hospitalizations	%	Deaths	HMR
Sex				
Male	293253	31.91	15667	5.34
Female	625699	68.09	29738	4.75
Age Group				
60–64 y	83444	9.8	1516	1.81
65–69 y	102666	11.17	2420	2.35
70–74 y	131867	14.35	3813	2.89
75–79 y	164476	17.90	6156	3.74
≥ 80 y	436499	47.50	31500	7.21
TOTAL	918952	100,00	45405	4.94

*Note:* HMR: In‐hospital case fatality rate (%), calculated as the ratio between the number of deaths and the number of hospital admissions, multiplied by 100. Data derived from the Hospital Information System of the Brazilian Unified Health System (SIH/SUS), 2008–2024.

Women accounted for the majority of hospital admissions (68.1%), whereas men exhibited a higher in‐hospital mortality rate compared with women (5.34% vs. 4.75%).

### 3.3. Hospital Costs

Between 2008 and 2024, total expenditure related to hospital admissions for femoral fractures among older adults in Brazil amounted to BRL 2.348 billion. Of this total, BRL 2.032 billion (86.5%) corresponded to direct hospital costs, while BRL 310.5 million (13.2%) was attributable to professional fees. A linear upward trend in total expenditures was observed over the study period, reflecting the increasing demand for care and potentially rising hospital costs and clinical complexity among patients.

The Southeast region accounted for the largest share of national expenditures, totaling BRL 1.213 billion (51.7%) of overall costs, followed by the South (BRL 476.6 million), Northeast (BRL 430.2 million), Midwest (BRL 138.0 million), and North (BRL 89.8 million) regions.

The Southeast also recorded the highest expenditures on professional fees (BRL 159.8 million), and the greatest absolute growth over the 2 decades analyzed. In contrast, the North region consistently exhibited the lowest expenditures across both cost categories, with a cumulative total of BRL 89.8 million over the study period.

From a temporal perspective, total hospital expenditures nearly quadrupled, increasing from BRL 61.2 million in 2008 to BRL 251.8 million in 2024, paralleling the rise in hospital admissions and in‐hospital mortality rates among the oldest age groups (≥ 80 years).

#### 3.3.1. Population‐Based In‐Hospital Mortality Rates per 100,000 Inhabitants

Age‐specific population‐based in‐hospital mortality rates due to femoral fractures remained low among individuals aged 60–64 years throughout the study period (2008–2024), with only modest regional variation. In 2024, rates ranged from 0.58 per 100,000 inhabitants in the Northeast to 1.44 per 100,000 in the Midwest. Over the entire period, the Southeast exhibited the highest mean rate (1.35 per 100,000), followed by the South (1.16) and the Midwest (0.95), whereas the lowest averages were observed in the North and the Northeast (0.59 and 0.58 per 100,000, respectively). The national mean rate was 0.93 per 100,000 inhabitants, indicating low magnitude and relative stability in this age group.

Among individuals aged 65–69 years, mortality rates increased compared with the younger group, with a national mean of 1.93 per 100,000 inhabitants between 2008 and 2024. In 2024, rates ranged from 1.17 per 100,000 in the North and Northeast to 2.94 per 100,000 in the Southeast. The Southeast maintained the highest mean rate over the study period (2.77 per 100,000), followed by the South (2.72) and the Midwest (1.84).

In the 70‐ to 74‐year age group, population‐based in‐hospital mortality rates continued to rise, with a national mean of 4.17 per 100,000 inhabitants over the study period. In 2024, rates varied from 3.09 per 100,000 in the North to 6.75 per 100,000 in the Southeast. The Southeast again presented the highest mean rate (5.97 per 100,000), followed by the South (5.67) and the Midwest (4.01).

A marked increase in mortality was observed among individuals aged 75–79 years, with a national mean rate of 9.94 per 100,000 inhabitants between 2008 and 2024. In the final year of the series, rates ranged from 7.31 per 100,000 in the Northeast to 14.68 per 100,000 in the Southeast. The Southeast exhibited the highest mean rate (13.82 per 100,000), followed by the South (13.34) and the Midwest (10.00). Temporal trends in this age group were characterized by intermittent increases and pronounced regional heterogeneity.

The highest mortality rates were observed among individuals aged 80 years and older. In this age group, all regions demonstrated statistically significant temporal trends (*p* < 0.0001), with a national mean rate of 49.11 per 100,000 inhabitants over the study period. In 2024, rates ranged from 35.75 per 100,000 in the Northeast to 78.68 per 100,000 in the South. The South consistently showed the highest mean rate (70.37 per 100,000), followed by the Southeast (60.70) and the Midwest (47.67). Temporal analysis revealed a sustained and pronounced increase, with wide interregional disparities, indicating consolidation of a high‐risk mortality profile among the oldest‐old population (*p* < 0.0001) (Table 3). Detailed cost distribution by region is presented in Table 4.

**TABLE 3:** Distribution of hospital, professional, and total costs associated with hospital admissions for femoral fractures among older adults in Brazil, by region (2008–2024).

Region	2008	2009	2010	2011	2012	2013	2014	2015	2016	2017	2018	2019	2020	2021	2022	2023	2024	Total
*Hospital Costs*																		
North	1.4	1.4	1.6	2.1	2.4	2.8	3.8	3.7	4.4	4.7	5.4	5.3	5.1	6.2	7.9	8.3	9.3	75.8
Northeast	9.1	10.7	10.9	12.1	11.8	14.8	16.3	16.4	17.8	20.7	24.5	27.4	27.6	31.0	37.5	40.7	40.7	370.0
Southeast	29.4	32.4	34.6	37.0	39.5	45.1	49.5	51.6	59.3	64.0	68.3	75.3	75.1	81.8	98.2	102.6	107.9	1051.5
South	9.7	11.0	12.5	13.6	14.3	18.4	19.9	22.3	24.7	25.1	28.3	29.4	30.3	33.2	39.5	41.3	43.2	416.6
Central West	2.7	3.2	3.5	3.2	3.8	4.2	4.6	5.5	6.3	7.1	8.1	9.2	8.4	8.7	11.5	13.9	14.4	118.2
TOTAL	52.2	58.8	63.1	67.9	71.8	85.3	94.1	99.4	112.6	121.5	134.6	146.6	146.5	160.8	194.6	206.7	215.5	2032.1

*Professional Costs*																		
North	0.3	0.3	0.3	0.4	0.5	0.5	0.6	0.6	0.8	0.9	0.9	1.0	0.9	1.3	1.5	1.5	1.7	13.8
Northeast	1.6	1.8	1.8	2.0	2.0	2.2	2.4	2.4	2.6	3.1	3.5	3.9	4.0	4.8	6.1	6.8	7.4	58.4
Southeast	5.1	5.6	5.9	6.2	6.6	6.9	7.5	7.8	9.0	9.6	10.0	10.8	10.8	11.8	14.5	15.3	16.3	159.8
South	1.6	1.8	2.0	2.1	2.2	2.6	2.8	3.2	3.5	3.5	3.8	3.9	4.1	4.5	5.5	5.8	6.1	59.1
Central West	0.5	0.6	0.6	0.6	0.7	0.7	0.8	0.9	1.0	1.2	1.3	1.5	1.4	1.4	1.8	2.2	2.3	19.3
TOTAL	9.0	10.0	10.6	11.3	11.9	12.8	14.1	14.8	17.0	18.3	19.6	21.1	21.2	23.8	29.4	31.7	33.8	310.5

*Total Costs*																		
North	1.6	1.7	1.9	2.5	2.9	3.3	4.3	4.2	5.2	5.5	6.4	6.3	6.0	7.5	9.4	9.9	11.2	89.8
Northeast	10.7	12.6	12.7	14.1	13.7	17.0	18.7	18.9	20.6	24.0	28.0	31.4	31.6	35.8	43.6	47.8	49.1	430.2
Southeast	34.5	38.0	40.6	43.2	46.1	52.0	57.1	59.5	68.4	73.8	78.6	86.3	85.9	93.6	112.8	118.2	125.1	1213.8
South	11.2	12.8	14.4	15.7	16.5	21.1	22.8	25.5	28.3	28.7	32.1	33.3	34.4	37.7	45.0	47.2	49.8	476.6
Central West	3.1	3.8	4.1	3.8	4.5	4.9	5.4	6.4	7.6	8.4	9.5	10.7	9.8	10.1	13.3	16.1	16.7	138.0
Overall	61.2	68.7	73.6	79.3	83.7	98.3	108.4	114.5	130.1	140.4	154.6	167.9	167.8	184.6	224.1	239.2	251.8	2348.3

*Note:* All values are expressed in millions of Brazilian reais (BRL million), as recorded in official health information systems.

**TABLE 4:** Population‐based in‐hospital mortality from femoral fractures among older adults in Brazil: Regional and age‐stratified analysis per 100,000 inhabitants (2008–2024).

Year	2008	2009	2010	2011	2012	2013	2014	2015	2016	2017	2018	2019	2020	2021	2022	2023	2024	Min–max	95% CI	*p* ‐value	Mean (SD)
*North*																					
60–64 y	0.62	0.29	1.41	0.27	0.51	1.44	0.69	0.43	0.41	0.20	0.19	0.55	0.70	0.51	0.65	0.47	0.76	0.19–1.44	[0.40; 0.78]	0.222	0.59 (±0.36)
65–69 y	0.41	1.18	1.89	0.73	1.05	1.00	0.96	0.31	1.16	1.37	1.30	1.24	1.18	0.45	2.39	2.49	0.79	0.31–2.49	[0.00; 2.38]	0.185	1.17 (±0.62)
70–74 y	2.23	2.14	1.54	1.48	2.38	3.21	4.85	2.97	2.04	4.30	2.62	2.51	2.40	1.64	3.13	3.56	3.09	1.48–4.85	[1.27; 4.91]	0.252	2.71 (±0.93)
75 a 79 y	5.00	7.21	5.37	8.07	7.01	5.35	5.76	8.57	5.88	6.21	7.59	8.86	6.04	3.91	7.57	9.52	10.81	3.91–10.81	[7.28; 14.34]	0.082	6.98 (±1.80)
≥ 80 y	17.85	16.59	24.42	31.63	29.82	35.54	29.34	23.54	25.84	22.07	25.58	22.52	23.64	24.54	30.09	39.97	37.39	16.59–39.97	[24.71; 50.07]	0.056	27.08 (±6.47)

*Northeast*																					
60–64 y	0.39	0.44	0.37	0.47	0.92	0.34	0.11	0.74	0.77	0.65	0.53	0.51	0.95	0.52	0.76	0.89	0.58	0.11–0.95	[0.13; 1.03]	0.0604	0.58 (±0.23)
65–69 y	0.50	0.57	0.95	1.46	0.89	1.56	0.96	1.25	0.96	1.36	1.39	0.88	1.42	1.33	1.35	1.51	1.51	0.50–1.56	[0.86; 2.16]	0.005	1.17 (±0.33)
70–74 y	0.96	1.64	2.38	2.13	2.00	2.33	2.65	1.33	2.15	2.47	2.69	2.20	2.42	3.93	3.97	2.77	4.59	0.96–4.59	[2.77; 6.41]	0.0003	2.51 (±0.93)
75 a 79 y	4.44	5.39	2.47	5.29	4.92	4.57	5.84	4.94	4.02	5.29	6.86	6.38	6.31	7.04	6.68	6.21	7.31	2.47–7.31	[4.86; 9.76]	0.0005	5.53 (±1.25)
≥ 80 y	12.70	20.21	16.57	22.41	20.11	19.04	22.18	21.48	21.89	25.38	26.57	28.47	30.21	34.32	33.10	32.70	35.75	12.70–35.75	[22.69; 48.81]	< 0.0001	24.89 (±6.66)

*Southeast*																					
60–64 y	0.94	1.31	1.22	1.42	1.21	1.05	1.31	1.13	1.27	1.85	1.25	1.38	1.39	1.36	1.68	1.82	1.35	0.94–1.85	[0.87; 1.83]	0.0126	1.35 (±0.24)
65–69 y	2.44	2.65	2.16	2.12	2.82	2.69	2.30	2.62	2.61	2.99	2.96	3.63	3.06	2.79	3.30	3.01	2.94	2.12–3.63	[2.17; 3.71]	0.0018	2.77 (±0.39)
70–74 y	5.23	6.16	4.91	4.58	5.32	6.23	5.22	4.68	6.25	7.22	6.70	6.54	6.53	7.64	6.19	5.33	6.75	4.58–7.64	[4.98; 8.52]	0.0202	5.97 (±0.90)
75 a 79 y	12.10	13.04	11.67	13.06	11.63	13.78	13.82	13.18	13.75	14.90	16.35	15.87	15.03	15.66	13.56	12.90	14.68	11.63–16.35	[11.90; 17.46]	0.0085	13.82 (±1.42)
≥ 80 y	51.10	51.63	50.58	47.35	51.00	54.03	53.82	57.88	65.62	66.31	68.43	70.70	67.88	72.74	67.07	66.55	69.23	47.35–72.74	[52.20; 86.26]	< 0.0001	60.70 (±8.69)

*South*																					
60–64 y	1.33	0.68	0.75	0.81	1.46	1.07	1.03	0.91	1.47	1.42	1.23	1.33	1.29	1.82	1.10	1.01	0.99	0.68–1.82	[0.40; 1.58]	0.2096	1.16 (±0.30)
65–69 y	3.82	2.51	2.67	1.59	2.68	3.67	2.54	2.02	3.47	2.49	2.12	2.38	2.37	3.40	2.99	2.81	2.72	1.59–3.82	[1.55; 3.89]	0.924	2.72 (±0.60)
70–74 y	6.10	4.71	5.08	4.12	4.64	6.05	5.98	7.61	7.13	7.57	5.09	4.83	6.62	4.74	5.49	4.86	5.71	4.12–7.61	[3.62; 7.80]	0.7928	5.67 (±1.07)
75 a 79 y	7.86	11.56	10.51	13.45	15.30	10.47	15.85	15.76	16.05	15.72	12.78	12.82	14.66	15.20	13.29	12.91	12.65	7.86–16.05	[8.13; 17.17]	0.1279	13.34 (±2.30)
≥ 80 y	50.51	54.64	57.60	69.44	61.13	71.73	72.10	83.25	83.39	80.91	82.90	78.04	78.61	81.42	86.11	77.08	78.68	50.51–86.11	[57.01; 100.35]	< 0.0001	73.38 (±11.06)

*Central West*																					
60–64 y	0.79	1.01	0.24	0.69	0.43	0.41	1.38	1.13	0.36	0.69	1.32	1.10	1.36	0.73	1.69	1.36	1.44	0.24–1.69	[0.57; 2.31]	0.0094	0.95 (±0.44)
65–69 y	1.76	0.68	2.29	1.57	1.50	0.29	1.64	2.34	2.22	3.28	2.01	2.76	1.42	2.72	2.05	1.79	1.03	0.29–3.28	[0.00; 2.51]	0.3428	1.84 (±0.76)
70–74 y	2.87	4.56	5.69	2.95	2.04	3.56	3.44	4.40	3.86	4.69	4.78	3.93	6.05	4.13	4.73	3.25	3.32	2.04–6.05	[1.30; 5.34]	0.4975	4.01 (±1.03)
75 a 79 y	6.65	11.28	6.70	5.72	7.83	7.99	12.49	11.44	10.50	8.69	16.32	11.22	11.23	11.24	10.78	10.63	9.36	5.72–16.32	[4.29; 14.43]	0.0592	10.00 (±2.59)
≥ 80 y	36.32	43.70	43.09	37.38	42.36	33.45	42.79	46.36	56.77	54.63	65.36	48.14	46.06	45.84	59.80	52.16	56.11	33.45–65.36	[39.02; 73.20]	0.0032	47.67 (±8.72)

*Brazil*																					
60–64 y	0.82	0.75	0.80	0.73	0.91	0.86	0.90	0.87	0.86	0.96	0.91	0.97	1.14	0.99	1.18	1.11	1.02	0.73–1.18	[0.77; 1.27]	< 0.0001	0.93 (±0.13)
65–69 y	1.78	1.52	1.99	1.49	1.79	1.84	1.68	1.71	2.08	2.30	1.96	2.18	1.89	2.14	2.42	2.32	1.80	1.49–2.42	[1.26; 2.34]	0.0058	1.93 (±0.27)
70–74 y	3.48	3.84	3.92	3.05	3.27	4.28	4.43	4.20	4.28	5.25	4.37	4.00	4.81	4.42	4.70	3.95	4.69	3.05–5.25	[3.58; 5.80]	0.0082	4.17 (±0.57)
75 a 79 y	7.21	9.69	7.34	9.12	9.34	8.43	10.75	10.78	10.04	10.16	11.98	11.03	10.66	10.61	10.38	10.43	10.96	7.21–11.98	[8.42; 13.50]	0.001	9.94 (±1.29)
≥ 80 y	33.70	37.35	38.45	41.64	40.89	42.76	44.05	46.50	50.70	49.86	53.77	49.58	49.28	51.77	55.23	53.69	55.43	33.70–55.43	[42.27; 68.59]	< 0.0001	46.74 (±6.71)
Total	7.77	8.58	8.17	8.58	8.73	9.19	9.38	9.72	10.55	10.99	11.22	11.16	11.06	11.65	11.29	10.84	11.40	7.77–11.40	[9.35; 10.71]	< 0.0001	10.03 (±1.32)

*Note:* Rates are expressed as population‐based in‐hospital mortality per 100,000 inhabitants aged ≥ 60 years. Mortality refers exclusively to deaths occurring during the index hospital admission. Mean values are presented with standard deviation (SD).

**FIGURE 1:**
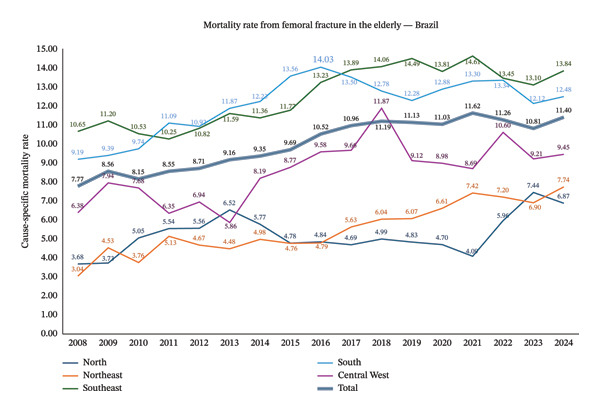
Population‐based in‐hospital mortality rates due to femoral fractures among individuals aged ≥ 60 years in Brazil, stratified by geographic macroregion, 2008–2024.

**FIGURE 2:**
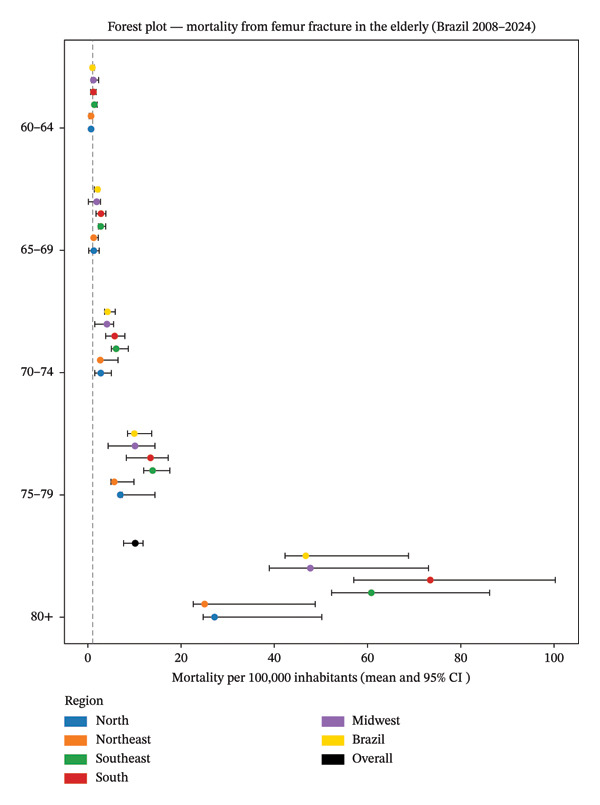
Forest plot 1—age‐stratified mortality from femoral fracture in the elderly: regional trends in Brazil (2008–2024).

Figure 1 illustrates annual population‐based in‐hospital mortality by region, showing a steady upward trend at the national level, increasing from 7.77 per 100,000 inhabitants in 2008 to 11.40 per 100,000 in 2024, highlighting the progressive worsening of in‐hospital mortality over time. The forest plot depicts age‐ and region‐specific in‐hospital mortality rates due to femoral fractures, expressed per 100,000 inhabitants with corresponding 95% confidence intervals. Mortality rates increased progressively with advancing age and remained relatively low among individuals aged 60–69 years, with overlapping confidence intervals across regions. From the age of 70 years onward, regional differences became more pronounced. The ≥ 80‐year age group exhibited the highest mean mortality rates, particularly in the South region, where rates exceeded 80 per 100,000 inhabitants, followed by the Southeast and Midwest regions (see Figure 2).

## 4. Discussion

Femoral fractures among older adults represent a major global public health problem, with severe functional, economic, social, and prognostic implications, particularly in countries undergoing rapid population aging [6, 9–11]. In the present study, 918,952 hospital admissions for femoral fractures among individuals aged 60 years and older were analyzed in Brazil between 2008 and 2024, during which more than 45,000 in‐hospital deaths were recorded.

### 4.1. Increase in Hospital Admission Rates

Several studies have demonstrated that population aging is the primary driver of the increasing rates of hospital admissions for femoral fractures among older adults worldwide [1, 2, 4], particularly in Brazil, where this trend reflects an accelerated demographic transition characterized by rapid growth of the older population and increased life expectancy [6, 10]. Between 2008 and 2024, the hospital admission rate for this condition increased by 41.7%, rising from 176.29 to 249.79 admissions per 100,000 inhabitants aged 60 years and older. This increase reflects not only a growth in the absolute number of hospitalizations but also structural changes in the country’s epidemiological profile and escalating demands on the healthcare system, which in 2024 served an older population of approximately 34 million individuals [1, 2, 9]. The increasing hospital admission rate reflects a growing hospital burden and does not necessarily indicate improvements in access to care; rather, it suggests a rising demand for hospitalizations that may be placing additional strain on existing healthcare resources [11, 12].

The incidence of femoral fractures is particularly high and more severe among the oldest‐old population [6, 10, 11]. This pattern can be explained by physiological changes inherent to aging, including loss of bone mineral density, sarcopenia, postural instability, and a higher prevalence of comorbid conditions. Von Friesendorff et al. [12] emphasize that the risk of mortality remains elevated even decades after the fracture event, particularly when associated with conditions such as pneumonia and heart failure.

Osteoporosis and osteopenia are central factors in this context, contributing significantly to the occurrence of femoral fractures [6, 9, 10]. However, osteoporosis remains largely underdiagnosed and undertreated in Brazilian primary healthcare, which further exacerbates fracture incidence [11]. Postmenopausal women constitute the most vulnerable group, given the accelerated decline in estrogen levels and its direct association with reduced bone mass [6, 9, 13, 14].

In addition, the presence of chronic diseases compromises the physiological response to trauma and surgery, thereby increasing the risk of complications and mortality. In the study by Machado et al. [6], which evaluated 395 older adults with proximal femoral fractures, several predictors of 1‐year in‐hospital mortality were identified, notably age ≥ 83 years, anemia, leukocytosis, and pulmonary infection. Patients presenting with pneumonia at admission exhibited a 5.6‐fold higher risk of death, whereas surgical delays exceeding 72 h between fracture and operative treatment nearly doubled mortality.

These findings reinforce that bone fragility cannot be dissociated from the overall clinical condition of older adults, with femoral fracture representing a terminal marker of multisystem decompensation. Integration across primary care, emergency services, and hospital‐based care becomes crucial to mitigate the impact of this condition, requiring multidisciplinary approaches that extend beyond isolated orthopedic management [15, 16].

### 4.2. In‐Hospital Mortality

Between 2008 and 2024, an overall in‐hospital mortality rate of 4.94% was observed for femoral fractures among older adults in Brazil, considering exclusively deaths occurring during the first hospital admission. This corresponds to approximately five deaths per 100 hospitalizations, underscoring the substantial clinical severity of this condition within the hospital setting. However, it is essential to emphasize that this estimate does not represent the total lethality associated with femoral fractures, as it does not capture deaths occurring after hospital discharge, during rehabilitation, or following readmissions due to late complications. This limitation is inherent to the administrative nature of SIH/SUS data, which do not allow for longitudinal postdischarge follow‐up.

Age‐stratified analysis revealed a clear and progressive age gradient in in‐hospital mortality, with a consistent increase as age advanced. The in‐hospital mortality rate ranged from 1.82% among individuals aged 60–64 years to 7.22% among the oldest‐old population (≥ 80 years), per 100 hospital admissions. This pattern reinforces the central role of senile frailty as a key clinical determinant of poorer prognosis, in line with findings from the international literature. von Friesendorff et al. demonstrated that mortality risk remains elevated for years following a hip fracture, particularly among the oldest individuals and in the presence of comorbidities, such as pneumonia, heart failure, and cognitive decline [12].

Regarding sex‐related differences, although women accounted for the majority of hospital admissions for femoral fractures during the study period (68.1%), men exhibited higher in‐hospital case fatality rates (5.34% vs. 4.75%). This finding is consistent with national and international evidence identifying male sex as an independent risk factor for mortality following femoral fracture. Machado et al. [6] identified male sex as a significant predictor of death, particularly among patients aged ≥ 80 years with multiple comorbidities. Similarly, Haleem et al. [15] reported higher mortality among men, which was attributed to a greater burden of chronic diseases, reduced physiological reserve, and poorer postoperative clinical trajectories.

When age‐adjusted population‐based in‐hospital mortality rates per 100,000 inhabitants were examined, the findings became even more pronounced among the oldest‐old population. The national mean mortality rate in this age group was 46.74 ± 6.71 deaths per 100,000 inhabitants, with values exceeding 66 per 100,000 in the Southeast and 59 per 100,000 in the Midwest. By comparison, age‐specific mortality increased from 0.82 per 100,000 inhabitants among individuals aged 60–64 years to 46.74 per 100,000 among those aged ≥ 80 years, representing an increase of more than 5600%. This rise did not occur linearly but intensified markedly after the age of 75 years, constituting a relevant epidemiological breakpoint consistent with the accentuation of senescence‐related processes and the progressive loss of functional reserve.

Regional analysis further revealed distinct patterns over the study period. While the Northeast region exhibited a 2.81‐fold increase in age‐specific population‐based in‐hospital mortality between 2008 and 2024, the South maintained consistently high rates with minimal variation (1.07‐fold), suggesting relative stabilization. The Midwest and North regions showed intermediate increases of 1.53‐ and 1.52‐fold, respectively. These findings indicate that, beyond population aging, mortality related to femoral fractures is strongly influenced by regional inequalities in healthcare organization, timely access to surgical treatment, and system capacity to respond to geriatric emergencies.

From a healthcare delivery and public policy perspective, these results underscore the need to incorporate age‐specific in‐hospital mortality due to femoral fractures among the oldest‐old as a key performance indicator of the healthcare system. Strategies such as early surgery (preferably within 48 h), multidisciplinary management, tailored protocols for frail patients, early rehabilitation, and fall‐prevention and osteoporosis screening programs are essential to mitigate the impact of this condition. In summary, advanced age emerges not merely as a statistical risk factor but as a high‐impact clinical prognostic marker, which should guide both healthcare planning and the formulation of public policies aimed at addressing the silent epidemic of femoral fractures among older adults in Brazil.

### 4.3. Regional Inequalities

The results of this study reveal marked regional disparities in the distribution of hospital admissions and outcomes related to femoral fractures among older adults in Brazil. The Southeast and South regions concentrated the highest absolute number of cases and also exhibited the highest population‐adjusted rates, contrasting with the lower values observed in the North and Northeast regions. These differences do not merely reflect demographic variation but also indicate structural inequalities in the organization, distribution, and access to referral hospital services.

In this context, previous studies indicate that economically more developed regions tend to concentrate a greater number of specialized professionals, high‐complexity hospitals, and adequate infrastructure for the management of geriatric orthopedic trauma. Such an organization facilitates more timely access to surgical treatment, a factor consistently recognized as crucial for reducing mortality following proximal femoral fractures [2, 6, 11, 16, 17]. Rojas et al. [18] emphasized that, in Latin America, the quality and consistency of hip fracture care vary widely across regions, including within Brazil [19], reinforcing the role of structural inequalities in shaping clinical outcomes.

Conversely, the data also reveal substantial regional disparities in mortality indicators. Studies conducted in the North and Northeast regions have identified significant limitations in access to referral centers, lower availability of multidisciplinary teams, and substantial delays in time to surgery [6, 10, 11]. The literature consistently demonstrates that surgical delays exceeding 72 h may double the risk of in‐hospital mortality, particularly among the oldest‐old population (≥ 80 years) and patients with multiple comorbidities, constituting a critical factor of healthcare vulnerability in these regions.

It is important to note, however, that the higher absolute concentration of deaths observed in the South and Southeast regions may also be partially explained by demographic factors. These regions have a higher proportion of oldest‐old individuals in the general population, which inherently increases the absolute risk of fractures and associated fatal outcomes. In contrast, the North and Northeast regions have relatively younger populations and lower life expectancy at birth, which reduces demographic exposure to the type of fracture analyzed, despite facing greater structural vulnerabilities in the provision of healthcare for older adults.

Accordingly, the regional findings of this study should be interpreted not only as epidemiological indicators but also as markers of social and structural inequality. Femoral fractures among older adults in Brazil thus represent a problem that extends beyond the clinical domain, requiring territorially tailored responses. Public health strategies should prioritize the regionalization of orthopedic care, strengthening of hospital networks, expansion of access to multidisciplinary teams, and improvement of equity in care delivery for the older population across all Brazilian macroregions.

### 4.4. Medical Expenditures

The results of this study demonstrate that, between 2008 and 2024, total expenditure on hospital admissions for femoral fractures among older adults in Brazil reached BRL 2.348 billion. Of this amount, BRL 2.032 billion (86.5%) corresponded to direct hospital costs, while BRL 310.5 million (13.2%) was attributable to professional fees. This distribution indicates that femoral fractures predominantly represent a high–resource‐consuming hospital event, involving prolonged hospital stays, surgical procedures, medical supplies, use of specialized inpatient beds, and continuous clinical support—a pattern consistent with that reported in international studies [19–21].

It is important to emphasize that these figures reflect only the costs of acute hospitalization and do not include expenditures related to rehabilitation, home care, long‐term institutionalization, or readmissions, which remain largely underestimated in the Brazilian context. In countries with more integrated health information systems, these additional costs are substantial. In the United Kingdom, Leal et al. demonstrated that 61% of total first‐year costs following hip fracture were concentrated in the index hospitalization, with a mean cost of £8613 per patient, within a total expenditure of £14,163 during the first year after hip fracture [20]. Similarly, in Ireland, Ferris et al. reported a mean cost of €11,700 per hospitalization episode, with a cumulative expenditure of €296 million between 2014 and 2020, in which acute hospitalization was the primary cost driver [21].

Temporal analysis revealed a marked increase in hospital expenditures in Brazil, rising from BRL 61.2 million in 2008 to BRL 251.8 million in 2024. According to IBGE [8], cumulative inflation between January 2008 and December 2024 was 159.94%. After adjustment for inflation, total hospital expenditures in 2024 correspond to approximately BRL 96.9 million in constant 2008 values, representing a real increase of about 58% over the study period.

These findings indicate that, although part of the observed growth reflects monetary correction, there remains a substantial real expansion in healthcare demand. This increase is likely driven by demographic aging, higher hospitalization rates, and greater clinical complexity among older adults, particularly those aged ≥ 80 years. Epidemiological evidence suggests that this age group experiences longer hospital stays, a higher incidence of complications, and an increase in in‐hospital mortality, all of which are associated with greater healthcare expenditures [22, 23].

The regional distribution of expenditures revealed a strong concentration in the Southeast region, which accounted for BRL 1.213 billion (51.7%) of total national spending, followed by the South (BRL 476.6 million) and Northeast (BRL 430.2 million) regions. This pattern parallels the higher proportion of older adults in these regions but also reflects a greater density of high‐complexity hospitals and higher hospitalization rates for femoral fractures. Similarly, international analyses have shown that regions with a greater supply of hospital beds and specialized services tend to exhibit higher absolute healthcare costs, regardless of age‐adjusted incidence rates [9, 24, 25].

The pattern observed in Brazil is consistent with the findings of the multicenter ICCONIC study, which evaluated 11 high‐income countries [26]. In that study, the United States reported the highest cost per hospitalization (USD 13,622 per admission) and the greatest total expenditure during the first year after fracture, largely driven by the intensive use of postfracture care services. In contrast, countries with more integrated universal healthcare systems, such as the Netherlands and England, demonstrated lower hospital costs, which were associated with shorter lengths of hospital stay and the organization of more efficient, structured care pathways [25].

In the national context, the mean costs of hospital admissions for femoral fractures exceeded those observed for hospitalizations due to neoplasms, infectious diseases, neurological disorders, and respiratory diseases [11], in addition to surpassing the combined costs associated with breast and gynecological cancers [22]. These findings underscore the disproportionate economic burden imposed by femoral fractures among older adults on the public healthcare system. Beyond the direct financial impact, hip fractures are associated with substantial functional and social consequences. It is estimated that approximately 40% of patients do not regain independent ambulation, while nearly 60% develop difficulties in basic activities of daily living, and up to 80% experience limitations in instrumental activities of daily living [22]. These outcomes significantly compromise quality of life among older adults and further amplify indirect costs by necessitating prolonged rehabilitation, home‐based care, or institutionalization, thereby intensifying the overall burden on healthcare and social support systems [24, 25].

### 4.5. Potential Biases

The results of this study should be interpreted in light of potential biases inherent to the ecological design and the use of secondary data. First, there is a possibility of information bias arising from underreporting, diagnostic coding errors, and regional inconsistencies in the completion of Hospital Admission Authorizations (Autorizações de Internação Hospitalar—AIH) within the SIH/SUS database. Such limitations may affect the absolute accuracy of the estimates; however, they are not expected to substantially compromise the temporal trends and regional patterns observed over the study period. Notwithstanding these limitations, this type of study design is widely employed in epidemiological surveillance and public health planning and is particularly suitable for large‐scale national analyses and long‐term time series.

Direct comparison of the population‐based in‐hospital mortality rate expressed per 100,000 inhabitants with findings from other studies was not feasible, as most available investigations report hip fracture mortality either as the proportion of deaths among hospitalized patients (in‐hospital case fatality) or as cumulative mortality at 30 days or 1 year, without the use of population denominators. The absence of this specific indicator in the literature limits direct population‐level comparisons; however, it does not compromise the assessment of internal temporal trends and regional disparities observed in the present study.

Finally, the inability to adjust for individual‐level clinical variables—such as fracture type, time to surgery, comorbidity severity, pre‐fracture functional status, or perioperative risk scores—may have introduced residual confounding. Nevertheless, the consistency of our findings with national and international literature suggests that the identified biases do not invalidate the results but should be taken into account when interpreting the outcomes.

## 5. Conclusion

Femoral fractures among older adults represent a major public health problem in Brazil, characterized by a high volume of hospital admissions, significant in‐hospital mortality, and progressively increasing healthcare costs that reflect both cumulative inflation and substantial real growth in healthcare demand. Between 2008 and 2024, a marked rise in hospitalizations and expenditures was observed, largely driven by population aging, increasing clinical complexity among patients, and persistent regional inequalities in access to healthcare services.

In‐hospital mortality was higher among the oldest‐old population and among men, reinforcing femoral fracture as an important marker of frailty and poor prognosis. These findings underscore the urgent need for strategies focused on prevention, early diagnosis of osteoporosis, timely surgical management, and appropriate rehabilitation to reduce the clinical and economic burden of femoral fractures in Brazil.

With more than BRL 2.3 billion spent over the study period, femoral fractures impose a substantial economic burden on the Brazilian public healthcare system, further exacerbated by fragmented care policies and the limited availability of multidisciplinary teams in regions distant from major urban centers. Strengthening primary healthcare, improving integrated care pathways for older adults, and reducing regional inequalities are fundamental measures to address this challenge in a more equitable and efficient manner.

In light of these findings, the results indicate that Brazil is still not fully prepared to confront the growing epidemic of femoral fractures among its older population. The sustained increase in hospital admissions, the high in‐hospital mortality among the oldest‐old, the escalating economic burden, and the marked regional inequalities reveal structural weaknesses in the organization of care pathways for older adults with femoral fractures. Accordingly, femoral fracture emerges as a sentinel event reflecting the capacity of the healthcare system to respond to accelerated population aging, reinforcing the urgent need for structural, integrated, and sustainable strategies to address this challenge in Brazil.

## Funding

No funding was received for this manuscript.

## Conflicts of Interest

The authors declare no conflicts of interest.

## Data Availability

The data that support the findings of this study are available from the corresponding author upon reasonable request.
